# Reproducibility and Comparability of Computational Models for Astrocyte Calcium Excitability

**DOI:** 10.3389/fninf.2017.00011

**Published:** 2017-02-21

**Authors:** Tiina Manninen, Riikka Havela, Marja-Leena Linne

**Affiliations:** Computational Neuroscience Group, Faculty of Biomedical Sciences and Engineering and BioMediTech Institute, Tampere University of TechnologyTampere, Finland

**Keywords:** reproducibility, comparability, astrocyte, calcium, computational model

## Abstract

The scientific community across all disciplines faces the same challenges of ensuring accessibility, reproducibility, and efficient comparability of scientific results. Computational neuroscience is a rapidly developing field, where reproducibility and comparability of research results have gained increasing interest over the past years. As the number of computational models of brain functions is increasing, we chose to address reproducibility using four previously published computational models of astrocyte excitability as an example. Although not conventionally taken into account when modeling neuronal systems, astrocytes have been shown to take part in a variety of *in vitro* and *in vivo* phenomena including synaptic transmission. Two of the selected astrocyte models describe spontaneous calcium excitability, and the other two neurotransmitter-evoked calcium excitability. We specifically addressed how well the original simulation results can be reproduced with a reimplementation of the models. Additionally, we studied how well the selected models can be reused and whether they are comparable in other stimulation conditions and research settings. Unexpectedly, we found out that three of the model publications did not give all the necessary information required to reimplement the models. In addition, we were able to reproduce the original results of only one of the models completely based on the information given in the original publications and in the errata. We actually found errors in the equations provided by two of the model publications; after modifying the equations accordingly, the original results were reproduced more accurately. Even though the selected models were developed to describe the same biological event, namely astrocyte calcium excitability, the models behaved quite differently compared to one another. Our findings on a specific set of published astrocyte models stress the importance of proper validation of the models against experimental wet-lab data from astrocytes as well as the careful review process of models. A variety of aspects of model development could be improved, including the presentation of models in publications and databases. Specifically, all necessary mathematical equations, as well as parameter values, initial values of variables, and stimuli used should be given precisely for successful reproduction of scientific results.

## 1. Introduction

Reproducibility of research results is a founding principle of scientific methodology. In general terms, it is defined as the ability of a study to be duplicated by any researcher. This dictates that all conditions affecting the original experimental setup must be known and reported. Reproducibility, reliability, and reuse of research results are becoming essential topics in the field of neuroscience.

In the field of computational neuroscience, computational models of brain function may not always contain all necessary information to reproduce the study, preventing the reuse of models in further studies (see, e.g., Cannon et al., 2007; De Schutter, 2008; Nordlie et al., 2009; Manninen et al., 2010; Crook et al., 2013; Stevens et al., 2013; Topalidou et al., 2015; Manninen et al., in press). Reproducibility of a modeling study describes how well the published simulation results can be produced by others, by implementing the model based on the information in the original publication, that is, not using any potentially available code (Crook et al., 2013; Cannon et al., 2014). Comparability, on the other hand, describes how well the published models can substitute one another. Reuse of models can also be hindered by the fact that models are often developed to describe specific neurophysiological phenomena and may not work properly in other research settings. As the number of computational models is increasing, it is important to carefully address the reproducibility, reuse, and comparability of models.

Theoretical insights from mathematical and computational models can make a valuable contribution to many different areas of neuroscience research, from modeling of molecular level biological processes to the analysis of large-scale patterns of brain activity. One emerging topic in the field of computational neuroscience is regulation of neuronal structure and function by glial cells. Relatively few data-driven, well-validated astrocyte models exist. This is partly because much of the data from astrocytes dates back to the 1990s, when most commonly used preparations were *in vitro* cell cultures and many modern experimental techniques had not yet been developed. This dictated the research hypotheses and questions asked. Moreover, the absence of signals comparable to neuronal action potentials is perhaps one of the main reasons why astrocytes have only recently attracted attention in the field of computational neuroscience. The controversial nature of experimental data related to astrocytes has slowed the progression of data-driven modeling in this field (see, e.g., Agulhon et al., 2010; Navarrete et al., 2013). Nevertheless, astrocytes express an overwhelming complexity of molecular and cell-level signaling and have been shown to interact with neurons in a variety of ways (see, e.g., recent review by Volterra et al., 2014). Therefore, as they are evidently shaping the neurophysiology and functioning of mammalian brains, it is necessary to address the principal astrocytic functions in future models of neural systems.

Several focused reviews of computational astrocyte models have appeared during the last few years (Jolivet et al., 2010; De Pittà et al., 2012; Fellin et al., 2012; Min et al., 2012; Volman et al., 2012; Wade et al., 2013; Linne and Jalonen, 2014; Tewari and Parpura, 2014; De Pittà et al., 2016). Some of these reviews discuss the involvement of astrocytes in normal physiological events in the brain, while some others concentrate on astrocytes' roles in the development of brain disorders and diseases. Some of the reviews also address astrocytes' potential roles in computation in the brain. Manninen et al. (in press) presented the first detailed categorization and evaluation of astrocyte-neuron models in a variety of neurophysiological functions. In this evaluation, more than 60 models were cataloged for astrocytes and astrocyte-neuron networks. To mention some examples, Höfer et al. (2002) and López-Caamal et al. (2014) have developed models for single astrocytes, Roth et al. (1995) and Bennett et al. (2008) for small astrocyte networks, Höfer et al. (2002) and Lallouette et al. (2014) for large astrocyte networks, Nadkarni and Jung (2003) and Tewari and Parpura (2013) for small astrocyte-neuron networks, and Allegrini et al. (2009) and Postnov et al. (2009) for large astrocyte-neuron networks. A detailed categorization of all existing models can be found in Manninen et al. (in press).

In our previous studies, we have assessed reproducibility and comparability issues in computational neuroscience and in computational cell biology (see, e.g., Pettinen et al., 2005; Manninen et al., 2010, 2011; Hituri and Linne, 2013; Manninen et al., in press). Especially in Manninen et al. (in press), we briefly discussed the reproducibility issues related to five astrocyte and astrocyte-neuron models (Nadkarni and Jung, 2003; Di Garbo et al., 2007; Lavrentovich and Hemkin, 2008; Dupont et al., 2011; Wade et al., 2012). We did not, however, address comparability in our previous work (Manninen et al., in press) as the emphasis was on categorization and general evaluation of all existing models. Here we aim to provide a systematic analysis of selected computational models for astrocyte functions, as part of our work to develop novel computational models for astrocyte research. We selected four relatively simple single astrocyte models to be implemented based on the information in the original publication (Lavrentovich and Hemkin, 2008; De Pittà et al., 2009; Dupont et al., 2011; Riera et al., 2011a,b). We tested if we were able to reproduce the original model behavior, especially the dynamical calcium (Ca^2+^) signals in astrocytes' somata, based on the information in the original publication. We also tested the comparability of the models by observing their dynamical behavior when the same stimulus or parameter values were used. We were especially interested in determining if these models could substitute one another when used as a module in a larger model. Our present study sheds light on functional differences between the models of astrocyte Ca^2+^ excitability. It also promotes reproducible science and development of good practices for publication of modeling results in the field of computational neuroscience.

## 2. Materials and methods

We compared models describing the two main types of astrocyte activity: spontaneous and neurotransmitter-evoked Ca^2+^ excitability. We performed selection of models for this study based on a large evaluation and characterization of more than 60 astrocyte Ca^2+^ activity models published by the end of 2014 (Manninen et al., in press), and exclusion criteria. We wanted to compare single astrocyte point models, and thus excluded models with diffusion and several cell components, such as astrocyte network, astrocyte-neuron interaction, or vascular interaction models. Most of the models are based on either the model by Li and Rinzel (1994) or the model by Höfer et al. (2002). Since it is not reasonable to compare models with the same core astrocyte Ca^2+^ activity model, only one of them was selected. The models selected based on these criteria were two models with spontaneous Ca^2+^ excitability (Lavrentovich and Hemkin, 2008; Riera et al., 2011a,b) and two models with neurotransmitter-evoked Ca^2+^ excitability (De Pittà et al., 2009; Dupont et al., 2011). The model by Lavrentovich and Hemkin (2008) is mainly based on the model by Höfer et al. (2002), and thus it was interesting to compare it to the model by Riera et al. (2011a,b) which is based on the models by Li and Rinzel (1994) and Höfer et al. (2002). The model by De Pittà et al. (2009) is mainly based on the model by Li and Rinzel (1994) with one reaction rate taken from the model by Höfer et al. (2002). It was compared to the model by Dupont et al. (2011) which is not based on the models by Li and Rinzel (1994) and Höfer et al. (2002) but represents its own line of astrocyte Ca^2+^ modeling.

Next, we present the models by Li and Rinzel (1994) and Höfer et al. (2002). These two models are used as basic building blocks in most existing models for astrocyte functions. It is therefore important to assess the nature of these models in order to perform reproducibility and comparability studies related to astrocyte models.

### 2.1. Model by Li and Rinzel (1994)

Li and Rinzel (1994) simplified the model by De Young and Keizer (1992). In the model by Li and Rinzel (1994), cytosolic Ca^2+^ concentration depends on Ca^2+^-induced Ca^2+^ release (CICR) from the endoplasmic reticulum (ER) to the cytosol, Ca^2+^ pump flux from the cytosol to the ER via sarco/ER Ca^2+^-ATPase (SERCA) pump, and leakage flux from the ER to the cytosol (leak ER). In the model by Li and Rinzel (1994), the differential equation for the Ca^2+^ concentration can be written as:

(1) $$\begin{array}{l} \frac{d{\left[Ca^{2+}\right]}_{cyt}}{dt}=\left(r_{CICR}m_{\infty }^{3}n_{\infty }^{3}h^{3}+r_{LEAK}\right)\\ \times \;\left({\left[Ca^{2+}\right]}_{free}-\left(1+c_{1}\right){\left[Ca^{2+}\right]}_{cyt}\right)\\ -\;V_{SERCA}\frac{{\left[Ca^{2+}\right]}_{cyt}^{2}}{{\left[Ca^{2+}\right]}_{cyt}^{2}+K_{SERCA}^{2}} \end{array}$$

and the differential equation for the fraction of active inositol 1,4,5-trisphosphate (IP_3_) receptors (IP_3_Rs) can be written as:

(2) $$\begin{array}{l} \frac{dh}{dt}=\frac{h_{\infty }-h}{\tau_{h}}, \end{array}$$

where

(3) $$\begin{array}{l} m_{\infty }=\frac{{\left[IP_{3}\right]}_{cyt}}{{\left[IP_{3}\right]}_{cyt}+d_{1}}, \end{array}$$

(4) $$\begin{array}{l} n_{\infty }=\frac{{\left[Ca^{2+}\right]}_{cyt}}{{\left[Ca^{2+}\right]}_{cyt}+d_{5}}, \end{array}$$

(5) $$\begin{array}{l} h_{\infty }=\frac{Q_{2}}{Q_{2}+{\left[Ca^{2+}\right]}_{cyt}}, \end{array}$$

(6) $$\begin{array}{l} \tau_{h}=\frac{1}{a_{2}\left(Q_{2}+{\left[Ca^{2+}\right]}_{cyt}\right)}, \end{array}$$

and

(7) $$\begin{array}{l} Q_{2}=d_{2}\frac{{\left[IP_{3}\right]}_{cyt}+d_{1}}{{\left[IP_{3}\right]}_{cyt}+d_{3}}. \end{array}$$

Li and Rinzel (1994) maintained IP_3_ concentration constant. The parameter values can be obtained from the literature (see, e.g., Li and Rinzel, 1994; De Pittà et al., 2009). Li and Rinzel (1994) also presented equations for Ca^2+^ efflux and influx across the plasma membrane when the total free Ca^2+^ concentration (${\left[Ca^{2+}\right]}_{free}$) was varying according to a differential equation.

### 2.2. Model by Höfer et al. (2002)

The model by Höfer et al. (2002) is based on several other publications (Atri et al., 1993; Dupont and Goldbeter, 1993; Höfer and Politi, 2001). They model up to 361 astrocytes and their model has four variables per astrocyte: cytosolic Ca^2+^ and IP_3_ concentrations, Ca^2+^ concentration in the ER, and fraction of active IP_3_Rs. The cytosolic Ca^2+^ concentration depends on CICR, leak ER, and SERCA pump across the ER membrane (*v*_Rel_ includes both CICR and leak ER) and Ca^2+^ efflux, influx, and leak across the plasma membrane (*v*_in_ includes both influx and leak), as well as diffusion of Ca^2+^ inside the cytosol and transfer of Ca^2+^ via gap junctions. The Ca^2+^ concentration in the ER depends on CICR, leak ER, and SERCA pump. The IP_3_ concentration depends on two distinct production terms via phospholipase C (PLC), one corresponding to PLCβ, which is activated through G-protein-coupled receptors exclusively in the stimulated cell, and the other to PLCδ, which is activated by Ca^2+^ elevation in the stimulated cell and in downstream cells, in addition to IP_3_ degradation, diffusion inside the cytosol, and transfer of IP_3_ via gap junctions. The fraction of active IP_3_Rs depends on rates for IP_3_R inactivation by Ca^2+^ binding and recovery. Thus, the model by Höfer et al. (2002) includes the following differential equations for the cytosolic Ca^2+^ concentration:

(8) $$\begin{array}{l} \frac{\partial {\left[Ca^{2+}\right]}_{cyt}}{\partial t}=v_{Rel}-v_{SERCA}+v_{in}-v_{out}\\ +\;D_{Ca}\left(\frac{\partial^{2}{\left[Ca^{2+}\right]}_{cyt}}{\partial x^{2}}+\frac{\partial^{2}{\left[Ca^{2+}\right]}_{cyt}}{\partial y^{2}}\right), \end{array}$$

for the Ca^2+^ concentration in the ER:

(9) $$\begin{array}{l} \frac{\partial {\left[Ca^{2+}\right]}_{ER}}{\partial t}=\beta \left(v_{SERCA}-v_{Rel}\right), \end{array}$$

for the IP_3_ concentration:

(10) $$\begin{array}{l} \;\frac{\partial {\left[IP_{3}\right]}_{cyt}}{\partial t}=v_{PLC\beta }+v_{PLC\delta }-v_{deg}\\ +\;D_{IP3}\left(\frac{\partial^{2}{\left[IP_{3}\right]}_{cyt}}{\partial x^{2}}+\frac{\partial^{2}{\left[IP_{3}\right]}_{cyt}}{\partial y^{2}}\right), \end{array}$$

and for the fraction of active IP_3_Rs:

(11) $$\begin{array}{l} \frac{\partial R}{\partial t}=v_{rec}-v_{inact}, \end{array}$$

where

(12) $$\begin{array}{l} v_{Rel}=\left(k_{1}+\frac{k_{2}R{\left[Ca^{2+}\right]}_{cyt}^{2}{\left[IP_{3}\right]}_{cyt}^{2}}{\left({\left[Ca^{2+}\right]}_{cyt}^{2}+K_{a}^{2}\right)\left({\left[IP_{3}\right]}_{cyt}^{2}+K_{IP3}^{2}\right)}\right)\\ \times \;\left({\left[Ca^{2+}\right]}_{ER}-{\left[Ca^{2+}\right]}_{cyt}\right), \end{array}$$

(13) $$\begin{array}{l} v_{SERCA}=k_{3}{\left[Ca^{2+}\right]}_{cyt}, \end{array}$$

(14) $$\begin{array}{l} v_{in}=v_{40}+v_{41}\frac{{\left[IP_{3}\right]}_{cyt}^{2}}{{\left[IP_{3}\right]}_{cyt}^{2}+K_{r}^{2}}, \end{array}$$

(15) $$\begin{array}{l} v_{out}=k_{5}{\left[Ca^{2+}\right]}_{cyt\;,} \end{array}$$

(16) $$\begin{array}{l} v_{PLC\delta }=v_{7}\frac{{\left[Ca^{2+}\right]}_{cyt}^{2}}{{\left[Ca^{2+}\right]}_{cyt}^{2}+K_{Ca}^{2}}, \end{array}$$

(17) $$\begin{array}{l} v_{PLC\beta }=v_{8}{\left(\left(1+\kappa_{G}\right)\left(\frac{\kappa_{G}}{1+\kappa_{G}}+\alpha_{0}\right)\right)}^{-1}\alpha_{0}, \end{array}$$

(18) $$\begin{array}{l} v_{deg}=k_{9}{\left[IP_{3}\right]}_{cyt}, \end{array}$$

and

(19) $$\begin{array}{l} v_{rec}-v_{inact}=k_{6}\left(\frac{K_{i}^{2}}{K_{i}^{2}+{\left[Ca^{2+}\right]}_{cyt}^{2}}-R\right). \end{array}$$

Equation (17) is given here as in the original publication since we were not able to verify it from any other source. Evidently, it could also be given in the form $v_{PLC\beta }=v_{8}{\left(\kappa_{G}+\left(1+\kappa_{G}\right)\alpha_{0}\right)}^{-1}\alpha_{0}$ which is much simpler and this raises a question if the equation was given incorrectly in the original publication. Most of the parameter values can be obtained from the literature (Höfer et al., 2002).

### 2.3. Single astrocyte models with spontaneous Ca^2+^ excitability

We implemented two single astrocyte models with spontaneous Ca^2+^ excitability. The first Ca^2+^ oscillation model was the model by Lavrentovich and Hemkin (2008), which is based on the models by Houart et al. (1999) and Höfer et al. (2002). The model by Lavrentovich and Hemkin (2008) is a generic model, that is not built to represent any specific brain area. However, they used some experimentally supported hypotheses to build their model (see, e.g., Parri et al., 2001; Aguado et al., 2002; Parri and Crunelli, 2003). The model includes three variables: Ca^2+^ concentration in the cytosol, Ca^2+^ concentration in the ER, and IP_3_ concentration (see Tables 1, 2). The second model was by Riera et al. (2011a,b), which is based on the models by Li and Rinzel (1994), Shuai and Jung (2002), Höfer et al. (2002), and Di Garbo et al. (2007). Riera et al. (2011a,b) included both modeling and wet-lab experimental work in mouse hippocampus. They used the experimental data to find the values for a few parameters. The model includes four variables: Ca^2+^ concentration, total free Ca^2+^ concentration, fraction of active IP_3_Rs, and IP_3_ concentration (see Tables 1, 3). In some of the simulations, Riera et al. (2011a) kept the total free Ca^2+^ concentration constant.

**Table 1:** **Model details**.

**Model**	**Model availability online**	**Graphical illustration given**	**Equations given**	**Stimuli given**	**Parameter values given**	**Initial conditions given**
De Pittà et al., 2009	No	Yes	Yes	Yes	Yes	No
Dupont et al., 2011	No	Yes	Yes	Yes	Yes	No
Lavrentovich and Hemkin, 2008	Yes	Yes	Yes	Spon.	Yes	Yes
Riera et al., 2011a,b	No	Yes	Yes	Spon.	Yes	No

*This table shows for four models how well the model details were given in the original publications. We reviewed the models based on several details: is the model available online, is a graphical illustration of the model given in the original publication, and are all the equations, stimuli, parameter values, and initial conditions given in the original publication. Spontaneous models we marked as “Spon.” under Stimuli. The model by Lavrentovich and Hemkin (2008) was found implemented in ModelDB, Accession number 112547. Errata were provided for two of the original publications (Lavrentovich and Hemkin, 2008; De Pittà et al., 2009). See Tables 2–5 for more details of what initial conditions we used if they were not given in the original publication*.

**Table 2:** **Details of the model by Lavrentovich and Hemkin (2008)**.

**Equation**	**Initial condition**	**Parameter value**
$\frac{d{\left[Ca^{2+}\right]}_{cyt}}{dt}=v_{in}-k_{out}{\left[Ca^{2+}\right]}_{cyt}+v_{CICR}-v_{SERCA}+k_{f}\left({\left[Ca^{2+}\right]}_{ER}-{\left[Ca^{2+}\right]}_{cyt}\right)$	0.1 μM	*k*_2_ = 0.1 μM
		*k*_CaA_ = 0.15 μM
$\frac{d{\left[Ca^{2+}\right]}_{ER}}{dt}=v_{SERCA}-v_{CICR}-k_{f}\left({\left[Ca^{2+}\right]}_{ER}-{\left[Ca^{2+}\right]}_{cyt}\right)$	1.5 μM	*k*_CaI_ = 0.15 μM
		*k*_deg_ = $0.08\frac{1}{s}$
$\frac{d{\left[IP_{3}\right]}_{cyt}}{dt}=v_{PLC}-k_{deg}{\left[IP_{3}\right]}_{cyt}$	0.1 μM	*k*_f_ = $0.5\frac{1}{s}$
		*k*_IP3_ = 0.1 μM
$v_{CICR}=4v_{M3}\frac{k_{CaA}^{n}{\left[Ca^{2+}\right]}_{cyt}^{n}}{\left({\left[Ca^{2+}\right]}_{cyt}^{n}+k_{CaA}^{n}\right)\left({\left[Ca^{2+}\right]}_{cyt}^{n}+k_{CaI}^{n}\right)}\frac{{\left[IP_{3}\right]}_{cyt}^{m}}{{\left[IP_{3}\right]}_{cyt}^{m}+k_{IP3}^{m}}\left({\left[Ca^{2+}\right]}_{ER}-{\left[Ca^{2+}\right]}_{cyt}\right)$		*k*_out_ = $0.5\frac{1}{s}$
		*k*_p_ = 0.3 μM
$v_{PLC}=v_{p}\frac{{\left[Ca^{2+}\right]}_{cyt}^{2}}{{\left[Ca^{2+}\right]}_{cyt}^{2}+k_{p}^{2}}$		*m* = 2.2
		*n* = 2.02
$v_{SERCA}=v_{M2}\frac{{\left[Ca^{2+}\right]}_{cyt}^{2}}{{\left[Ca^{2+}\right]}_{cyt}^{2}+k_{2}^{2}}$		*v*_in_ = $0.05\frac{\mu M}{s}$
		*v*_M2_ = $15\frac{\mu M}{s}$
		*v*_M3_ = $40\frac{1}{s}$
		*v*_p_ = $0.05\frac{\mu M}{s}$

*This table shows the original equations, parameter values, and initial conditions given in the original publication. Lavrentovich and Hemkin (2008) were the only ones who presented all the values in the original publication. Some of the parameter values that they modified in their simulations were, however, presented wrongly and a corrigendum was provided. The model has three variables: cytosolic Ca^2+^ concentration (${\left[Ca^{2+}\right]}_{cyt}$), Ca^2+^ concentration in the ER (${\left[Ca^{2+}\right]}_{ER}$), and cytosolic IP_3_ concentration ([I_P_3_]cyt_)*.

**Table 3:** **Details of the model by Riera et al. (2011a,b)**.

**Equation**	**Initial condition**	**Parameter value**
$\frac{d{\left[Ca^{2+}\right]}_{cyt}}{dt}=v_{Rel}-v_{SERCA}+\epsilon \left(j_{in}+v_{CCE}-v_{out}\right)$	0.09 μM	*a* = 0.2 $\frac{1}{\mu Ms}$
		*c*_1_ = 0.185
$\frac{d{\left[Ca^{2+}\right]}_{free}}{dt}=\epsilon \left(j_{in}+v_{CCE}-v_{out}\right)$	2 μM	*d*_1_ = 0.13 μM
		*d*_2_ = 1.049 μM
Original: $\frac{dh}{dt}=\alpha_{h}\left(1-h\right)+\beta_{h}h$		*d*_3_ = 0.9434 μM
		*d*_5_ = 0.082 μM
Modified: $\frac{dh}{dt}=\alpha_{h}\left(1-h\right)-\beta_{h}h$	0.79	ϵ = 0.01
		*H*_CCE_ = 10 μM
$\frac{d{\left[IP_{3}\right]}_{cyt}}{dt}=X_{IP3}+PLC_{\delta 1}-K_{IP3}{\left[IP_{3}\right]}_{cyt}$	0.14 μM	*j*_in_ = $0.065\frac{\mu M}{s}$
		*K*_IP3_ = $1.25\frac{1}{s}$
$\alpha_{h}=ad_{2}\frac{{\left[IP_{3}\right]}_{cyt}+d_{1}}{{\left[IP_{3}\right]}_{cyt}+d_{3}}$		*K*_δCa_ = 0.55 μM
		*k*_out_ = $0.5\frac{1}{s}$
$\beta_{h}=a{\left[Ca^{2+}\right]}_{cyt}$		*K*_p_ = 0.1 μM
		*v*_1_ = $6\frac{1}{s}$
${\left[Ca^{2+}\right]}_{ER}=\frac{{\left[Ca^{2+}\right]}_{free}-{\left[Ca^{2+}\right]}_{cyt}}{c_{1}}$		*v*_2_ = $0.11\frac{1}{s}$
		*v*_δ_ = $0.152\frac{\mu M}{s}$
$m_{\infty }=\frac{{\left[IP_{3}\right]}_{cyt}{\left[Ca^{2+}\right]}_{cyt}}{\left({\left[IP_{3}\right]}_{cyt}+d_{1}\right)\left({\left[Ca^{2+}\right]}_{cyt}+d_{5}\right)}$		*V*_SERCA_ = $0.9\frac{\mu M}{s}$
		*x*_CCE_ = $0.01\frac{\mu M}{s}$
$PLC_{\delta 1}=v_{\delta }\frac{{\left[Ca^{2+}\right]}_{cyt}^{2}}{{\left[Ca^{2+}\right]}_{cyt}^{2}+K_{\delta Ca}^{2}}$		*X*_IP3_ = $0.43\frac{\mu M}{s}$
$v_{CCE}=x_{CCE}\frac{H_{CCE}^{2}}{H_{CCE}^{2}+{\left[Ca^{2+}\right]}_{ER}^{2}}$		
$v_{out}=k_{out}{\left[Ca^{2+}\right]}_{cyt}$		
$v_{Rel}=c_{1}\left(v_{1}m_{\infty }^{3}h^{3}+v_{2}\right)\left({\left[Ca^{2+}\right]}_{ER}-{\left[Ca^{2+}\right]}_{cyt}\right)$		
$v_{SERCA}=V_{SERCA}\frac{{\left[Ca^{2+}\right]}_{cyt}^{2}}{{\left[Ca^{2+}\right]}_{cyt}^{2}+K_{p}^{2}}$		

**Figure 1:**
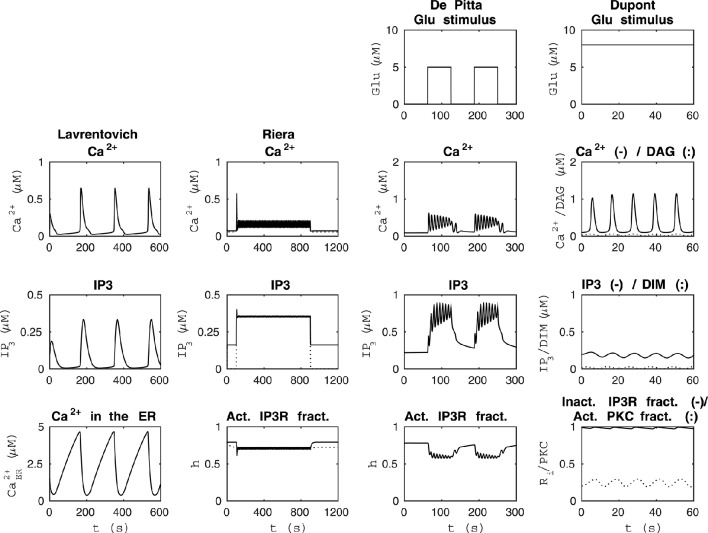
**Reproducibility of the basic model behavior with the original parameter values and stimulus**. The first column from the left presents the simulation of the model by Lavrentovich and Hemkin (2008) in the same condition as Figure 3 of the original publication except that the concentrations of IP_3_ and Ca^2+^ in the ER were not plotted in the original publication (see Table 2). The second column from the left shows simulation results of our modified version of the model by Riera et al. (2011a) (see Table 3) when the total free Ca^2+^ concentration was set to a constant value of 2 μM (*j*_in_+*v*_CCE_−*v*_OUT_ = 0) based on Figure 4b of the original publication and *X*_IP3_ was a pulse function. Thus, *X*_IP3_ was 0.43 μM/s between 100 and 900 s and either 0 (curves with dotted lines) or 0.2 μM/s (curves with solid lines) otherwise. The second column from the right shows simulation results in the same condition as Figure 12 AM of the original publication by De Pittà et al. (2009) (stimulus was a two-pulse wave with alternating Glu concentrations of 2 nM and 5 μM, pulse duration of 62.5 s, and period 125 s), except that the fraction of active IP_3_Rs (*h*) was not plotted in the original publication (see Table 4). The first column from the right shows simulation results of our modified version of the model by Dupont et al. (2011) in the same condition as Figure 2 of the original publication by Dupont et al. (2011) (stimulus was a constant Glu concentration of 8 μM), except that the IP_3_ concentration and fraction of inactive IP_3_Rs (*R*_i_) were not plotted in the original publication (see Table 5). See Table 6 for more details.

*This table shows the original equations and parameter values given in the original publication as well as our modified version of one of the differential equations and our values for the initial conditions since Riera et al. (2011a,b) did not give the initial conditions. We did not take into account the stochastic terms in the original differential equations. The model has four variables: cytosolic Ca^2+^ concentration (${\left[Ca^{2+}\right]}_{cyt}$), free total Ca^2+^ concentration (${\left[Ca^{2+}\right]}_{free}$), fraction of active IP_3_Rs (h), and cytosolic IP_3_ concentration ([I_P_3_]cyt_). The modified equation for h here is just a different way to write Equation (2). Riera et al. (2011a,b) initiated their simulation with a pulse of X_IP3_ as explained in Figure 1. However, only the value during the pulse was clearly given in the original publication and not the initial value*.

### 2.4. Single astrocyte models with neurotransmitter-evoked Ca^2+^ excitability

We implemented two single astrocyte models with neurotransmitter-evoked Ca^2+^ excitability. The first one was the generic model by De Pittà et al. (2009) for glutamate (Glu)-induced astrocytic Ca^2+^ dynamics, which is based on the models by De Young and Keizer (1992), Li and Rinzel (1994), and Höfer et al. (2002). Several key observations on a variety of cell types were used to construct the model, e.g., IP_3_ kinetics data from Xenopus oocytes. De Pittà et al. (2009) also used experimental data by Tsodyks and Markram (1997) as input to their model. The model by De Pittà et al. (2009) includes three model variables: Ca^2+^ concentration, IP_3_ concentration, and fraction of active IP_3_Rs (see Tables 1, 4). De Pittà et al. (2009) pointed out in their publication that *h* denotes fraction of inactive IP_3_Rs. However, they took the variable *h* from the model by Li and Rinzel (1994) where *h* is used to describe fraction of active IP_3_Rs. The second model was the generic model by Dupont et al. (2011) for metabotropic Glu receptor 5 (mGlu5R)-induced Ca^2+^ oscillations. The model is based on their previous models (Dupont and Goldbeter, 1993; Dupont and Croisier, 2010), and they compared their simulation results with some experimental data from, e.g., Chinese hamster ovary cells (Nash et al., 2002). Their model includes six variables: Ca^2+^ concentration, diacylglycerol (DAG) concentration, ligand-bound mGlu5R dimer (DIM) concentration, IP_3_ concentration, fraction of active protein kinase C (PKC), and fraction of Ca^2+^-inhibited IP_3_Rs meaning fraction of inactive IP_3_Rs (see Tables 1, 5).

**Table 4:** **Details of the model by De Pittà et al. (2009)**.

**Equation**	**Initial condition**	**Parameter value**
$\frac{d{\left[Ca^{2+}\right]}_{cyt}}{dt}=J_{chan}+J_{leak}-J_{pump}$	0.09 μM	*a*_2_ = $0.2\frac{1}{\mu Ms}$
		*c*_1_ = 0.185
$\frac{dh}{dt}=\frac{h_{\infty }-h}{\tau_{h}}$	0.78	${\left[Ca^{2+}\right]}_{free}$ = 2 μM
		*d*_1_ = 0.13 μM
$\frac{d{\left[IP_{3}\right]}_{cyt}}{dt}=v_{glu}+v_{\delta }-v_{3K}-{\bar{r}}_{5P}{\left[IP_{3}\right]}_{cyt}$	0.22 μM	*d*_2_ = 1.049 μM
		*d*_3_ = 0.9434 μM
$h_{\infty }=\frac{Q_{2}}{Q_{2}+{\left[Ca^{2+}\right]}_{cyt}}$		*d*_5_ = 0.08234 μM
		κ_δ_ = 1.5 μM
$J_{chan}=r_{C}m_{\infty }^{3}n_{\infty }^{3}h^{3}\left({\left[Ca^{2+}\right]}_{free}-\left(1+c_{1}\right){\left[Ca^{2+}\right]}_{cyt}\right)$		*K*_3_ = 1 μM
		*K*_π_ = 0.6 μM
$J_{leak}=r_{L}\left({\left[Ca^{2+}\right]}_{free}-\left(1+c_{1}\right){\left[Ca^{2+}\right]}_{cyt}\right)$		*K*_D_ = 0.7 μM
		*K*_ER_ = 0.1 μM
$J_{pump}=v_{ER}\frac{{\left[Ca^{2+}\right]}_{cyt}^{2}}{{\left[Ca^{2+}\right]}_{cyt}^{2}+K_{ER}^{2}}$		*K*_p_ = 10 μM
		*K*_PLCδ_ = 0.1 μM
$K_{\gamma }=K_{R}\left(1+\frac{K_{p}}{K_{R}}\frac{{\left[Ca^{2+}\right]}_{cyt}}{{\left[Ca^{2+}\right]}_{cyt}+K_{\pi }}\right)$		*K*_R_ = 1.3 μM
		${\bar{r}}_{5P}$ = $0.04\frac{1}{s}$
$m_{\infty }=\frac{{\left[IP_{3}\right]}_{cyt}}{{\left[IP_{3}\right]}_{cyt}+d_{1}}$		*r*_C_ = $6\frac{1}{s}$
		*r*_L_ = $0.11\frac{1}{s}$
$n_{\infty }=\frac{{\left[Ca^{2+}\right]}_{cyt}}{{\left[Ca^{2+}\right]}_{cyt}+d_{5}}$		${\bar{v}}_{3K}$ = $2\frac{\mu M}{s}$
		${\bar{v}}_{\beta }$ = $0.2\frac{\mu M}{s}$
$Q_{2}=d_{2}\frac{{\left[IP_{3}\right]}_{cyt}+d_{1}}{{\left[IP_{3}\right]}_{cyt}+d_{3}}$		${\bar{v}}_{\delta }$ = $0.02\frac{\mu M}{s}$
		*v*_ER_ = $0.9\frac{\mu M}{s}$
$\tau_{h}=\frac{1}{a_{2}\left(Q_{2}+{\left[Ca^{2+}\right]}_{cyt}\right)}$		
$v_{3K}={\bar{v}}_{3K}\frac{{\left[Ca^{2+}\right]}_{cyt}^{4}}{{\left[Ca^{2+}\right]}_{cyt}^{4}+K_{D}^{4}}\frac{{\left[IP_{3}\right]}_{cyt}}{{\left[IP_{3}\right]}_{cyt}+K_{3}}$		
$v_{\delta }=\frac{{\bar{v}}_{\delta }}{1+\frac{{\left[IP_{3}\right]}_{cyt}}{\kappa_{\delta }}}\frac{{\left[Ca^{2+}\right]}_{cyt}^{2}}{{\left[Ca^{2+}\right]}_{cyt}^{2}+K_{PLC\delta }^{2}}$		
$v_{glu}={\bar{v}}_{\beta }\frac{{\left[Glu\right]}_{syn}^{0.7}}{{\left[Glu\right]}_{syn}^{0.7}+K_{\gamma }^{0.7}}$		

*This table shows the original equations and parameter values for AM case given in the original publication as well as our initial conditions since De Pittà et al. (2009) did not give the initial conditions. De Pittà et al. (2009) had an error in the unit of parameter a_2_. The model has three variables: cytosolic Ca^2+^ concentration (${\left[Ca^{2+}\right]}_{cyt}$), fraction of active IP_3_Rs (h), and cytosolic IP_3_ concentration ([I_P_3_]cyt_). De Pittà et al. (2009) stimulated their model with a two-pulse wave of Glu as explained in Figure 1. In this table, we have marked Glu stimulus γ by De Pittà et al. (2009) as [Glu]_syn_*.

**Table 5:** **Details of the model by Dupont et al. (2011)**.

**Equation**	**Initial condition**	**Parameter value**
Original: $\frac{d{\left[Ca^{2+}\right]}_{cyt}}{dt}=v_{0}+k_{i}\left(b_{1}+IR_{a}\right)-V_{MP}\frac{{\left[Ca^{2+}\right]}_{cyt}^{2}}{{\left[Ca^{2+}\right]}_{cyt}^{2}+K_{P}^{2}}-k_{l}{\left[Ca^{2+}\right]}_{cyt}$		α = 0.1
		*b*_1_*k*_i_ = $7.5\times 10^{-4}\frac{\mu M}{s}$
Modified: $\frac{d{\left[Ca^{2+}\right]}_{cyt}}{dt}=v_{0}+k_{i}\left(b_{1}+IR_{a}\right)\left({\left[Ca^{2+}\right]}_{tot}-\left(\alpha +1\right){\left[Ca^{2+}\right]}_{cyt}\right)-V_{MP}\frac{{\left[Ca^{2+}\right]}_{cyt}^{2}}{{\left[Ca^{2+}\right]}_{cyt}^{2}+K_{P}^{2}}-k_{l}{\left[Ca^{2+}\right]}_{cyt}$	0.1 μM	${\left[Ca^{2+}\right]}_{tot}$ = 80 μM
		*k*_1_ = $0.12\frac{1}{s}$
$\frac{d{\left[DAG\right]}_{cyt}}{dt}=k_{PLC}DIM-V_{MD}\frac{{\left[DAG\right]}_{cyt}}{{\left[DAG\right]}_{cyt}+K_{MD}}$	25 × 10^−3^ μM	*K*_A_ = 5 × 10^−4^ μM
		*K*_A1_ = 5 × 10^−4^ μM
$\frac{dDIM}{dt}=V_{M1}\frac{DIM^{P}}{DIM^{P}+K_{A1}}-V_{PKC}PKC\frac{DIM}{DIM+K_{A}}$	14 × 10^−3^ μM	*k*_act_ = $0.2\frac{1}{s}$
		*K*_act_ = 0.34 μM
$\frac{d{\left[IP_{3}\right]}_{cyt}}{dt}=k_{PLC}DIM-k_{1}{\left[IP_{3}\right]}_{cyt}$	0.2 μM	*K*_AD_ = 0.06 μM
		*K*_aff_ = 2 μM^2^
$\frac{dPKC}{dt}=k_{act}\frac{{\left[DAG\right]}_{cyt}}{{\left[DAG\right]}_{cyt}+K_{AD}}\left(1-PKC\right)-k_{des}PKC$	0.2	*k*_des_ = $0.2\frac{1}{s}$
		*k*_di_ = 0.1 μM
$\frac{dR_{i}}{dt}=k_{i+}{\left[Ca^{2+}\right]}_{cyt}^{4}\left(1-R_{i}\right)\frac{K_{act}^{3}}{K_{act}^{3}+{\left[Ca^{2+}\right]}_{cyt}^{3}}-k_{i-}R_{i}$	0.9898	*k*_i_ = $7.5\frac{\mu M}{s}$
		*K*_I_ = 0.4 μM
$DIM^{P}=\frac{R_{tot}-\sqrt{K_{di}R_{2}}-2R_{2}-2DIM}{2}$		*k*_i+_ = $25\frac{1}{\mu M^{4}s}$
		*k*_i−_ = $0.0025\frac{1}{s}$
$IR_{a}=\left(1-R_{i}\right)\frac{{\left[IP_{3}\right]}_{cyt}^{2}}{{\left[IP_{3}\right]}_{cyt}^{2}+K_{I}^{2}}\frac{{\left[Ca^{2+}\right]}_{cyt}^{3}}{{\left[Ca^{2+}\right]}_{cyt}^{3}+K_{act}^{3}}$		*k*_l_ = $0.0025\frac{1}{s}$
		*K*_MD_ = 0.012 μM
$R_{2}=K_{a\mathrm{ff}}\frac{DIM}{{\left[Glu\right]}_{syn}^{2}}$		*K*_P_ = 0.4 μM
		*k*_PLC_ = $1.25\frac{1}{s}$
		*R*_tot_ = 0.075 μM
		*v*_0_ = $0.025\frac{\mu M}{s}$
		*V*_M1_ = $0.05\frac{\mu M}{s}$
		*V*_MD_ = $0.0325\frac{\mu M}{s}$
		*V*_MP_ = $2\frac{\mu M}{s}$
		*V*_PKC_ = $0.2\frac{\mu M}{s}$

*This table shows the original equations and parameter values as well as our initial conditions since Dupont et al. (2011) did not give the initial conditions. With the original equations by Dupont et al. (2011), we were not able to obtain Ca^2+^ oscillations as presented by Dupont et al. (2011). Thus, we made modifications to the model by Dupont et al. (2011) using the model by Dupont and Croisier (2010) (parameters b_1_k_i_ and k_i_ have now same values as the original values but different unit 1/s compared to the original unit μM/s). The model has six variables: cytosolic Ca^2+^ concentration (${\left[Ca^{2+}\right]}_{cyt}$), cytosolic DAG concentration ([DAG]), concentration of ligand-bound mGlu5R dimers (DIM), cytosolic IP_3_ concentration ([I_P_3_]cyt_), fraction of active protein kinase C (PKC), and fraction of Ca^2+^-inhibited IP_3_Rs (R_i_). Dupont et al. (2011) stimulated their model with a constant Glu concentration as explained in Figure 1. In this table, we have marked Glu stimulus L by Dupont and Croisier (2010) as [Glu]_syn_*.

### 2.5. Simulations

**Figure 2:**
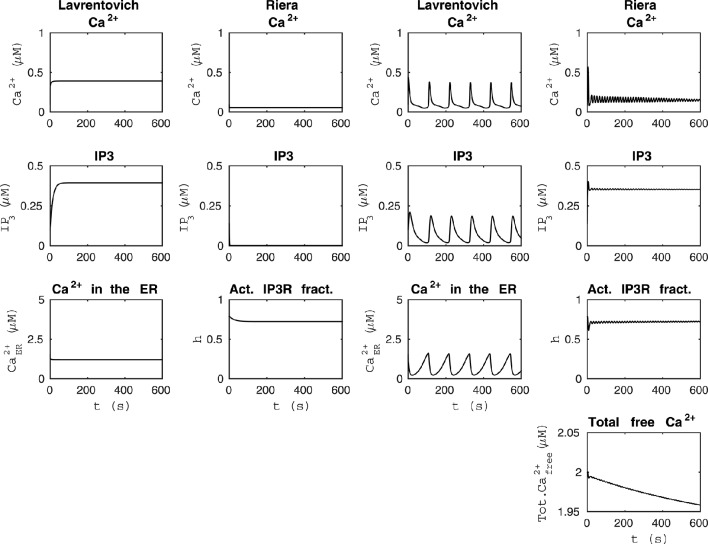
**Comparability of the models by Lavrentovich and Hemkin (2008) and Riera et al. (2011a,b)**. The first column from the left shows that the model by Lavrentovich and Hemkin (2008) did not oscillate when the sum of fluxes over the cell membrane was zero ($v_{in}-k_{out}\left[Ca^{2+}\right]=0$) and otherwise similar simulation setup as in Figure 1. The second column from the left shows that our modified version of the model by Riera et al. (2011a,b) did not oscillate when we changed the parameter producing IP_3_ (*X*_IP3_) to zero in addition to having the total free Ca^2+^ concentration as constant value of 2 μM and otherwise similar simulation setup as in Figure 1. In this case, IP_3_ concentration is almost zero. The second column from the right shows the simulation results of the model by Lavrentovich and Hemkin (2008) when we changed the value of *V*_M2_ to 5.8 μM/s and otherwise similar simulation setup as in Figure 1. The first column from the right shows the simulation results of the model by Riera et al. (2011a,b) when we had total free Ca^2+^ concentration as a variable, *X*_IP3_ as 0.43 μM/s, and otherwise similar simulation setup as in Figure 1. The dynamical behaviors of these models were still different.

**Figure 3:**
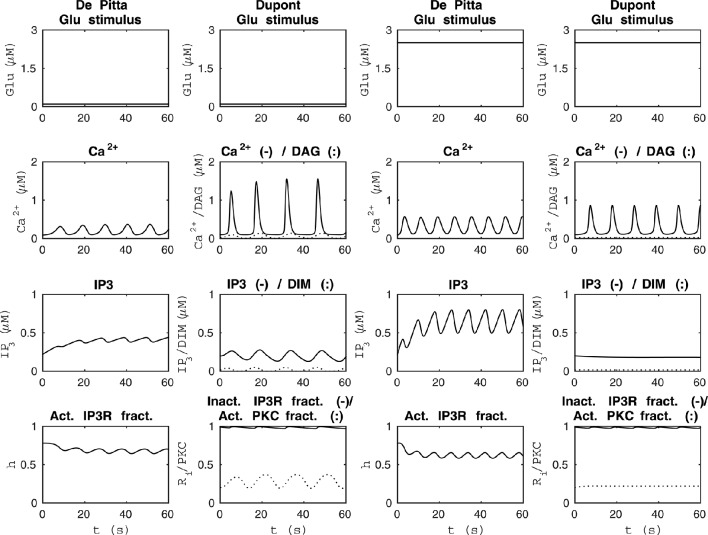
**Comparability of the model by De Pittà et al. (2009) and our modified version of the model by Dupont et al. (2011) with two different constant Glu stimuli**. The two columns from the left show how the models behaved when the stimulus was a constant Glu concentration of 0.1 μM. The two columns from the right show how the models behaved when the stimulus was a constant Glu concentration of 2.5 μM. The models had opposite behaviors with these two specific stimuli; the higher constant stimulus value produced higher Ca^2+^ concentrations with the model by De Pittà et al. (2009), whereas it produced lower Ca^2+^ concentrations with our modified version of the model by Dupont et al. (2011).

**Figure 4:**
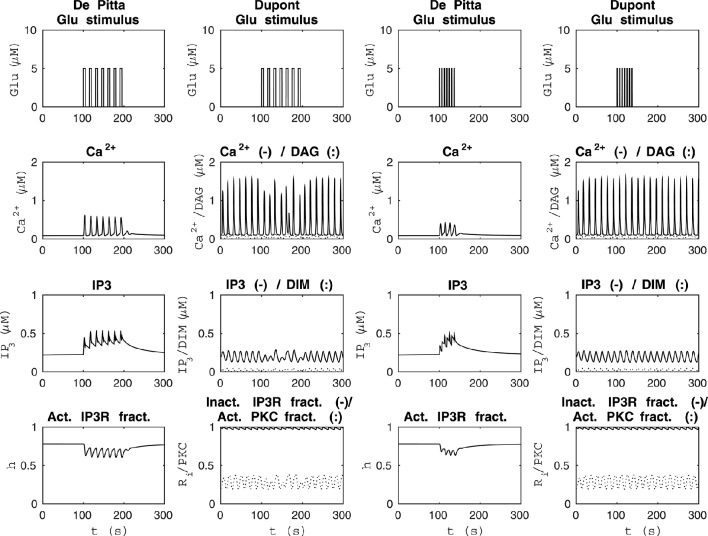
**Comparability of the model by De Pittà et al. (2009) and our modified version of the model by Dupont et al. (2011) with two different seven-pulse waves of Glu stimulus**. The two columns from the left show how the models behaved when the stimulus was a seven-pulse wave with alternating Glu concentrations of 2 nM and 5 μM, pulse duration of 5 s, and period 15 s. The two columns from the right show how the models behaved when the stimulus was a seven-pulse wave with alternating Glu concentrations of 2 nM and 5 μM, pulse duration of 1 s, and period 6 s. With our modified version of the model by Dupont et al. (2011), the Ca^2+^ concentration oscillated even at Glu stimulus of 2 nM, which was not the case with the model by De Pittà et al. (2009).

**Figure 5:**
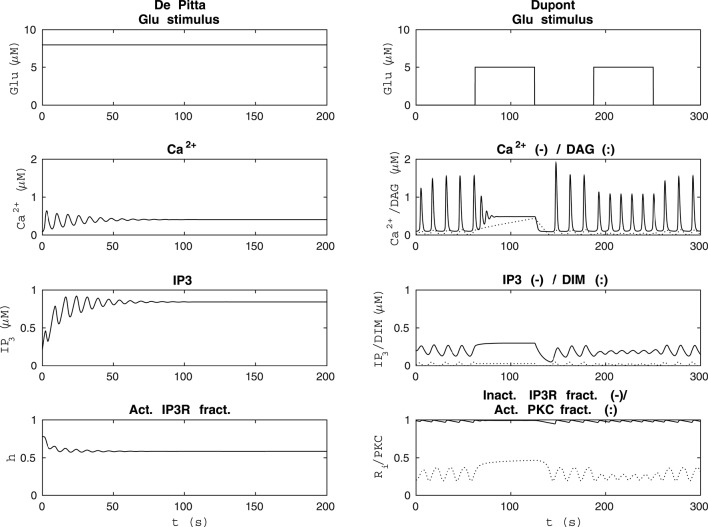
**Comparability of the model by De Pittà et al. (2009) and our modified version of the model by Dupont et al. (2011) with each other's original stimulus**. The left column shows the results of the model by De Pittà et al. (2009) when the stimulus was a constant Glu concentration of 8 μM (the original stimulus of the model by Dupont et al., 2011). The model by De Pittà et al. (2009) ceased to oscillate around 100 s. The right column shows how the original stimulus from the model by De Pittà et al. (2009) (a two-pulse wave with alternating Glu concentrations of 2 nM and 5 μM, pulse duration of 62.5 s, and period 125 s) affected our modified version of the model by Dupont et al. (2011).

We implemented the models in MATLAB® and in Python based on the information in the original publications, such as equations, parameter values, initial conditions, and stimuli (see Tables 1–5), and simulated the models. In MATLAB®, we used both the forward Euler method and built-in differential equation solvers, such as ode15s. In Python, we built and ran the models using Jupyter Notebook (jupyter.org) and used Scipy's differential equation solver odeint. Simulations run using different platforms and solvers produced consistent results. The models implemented in Python can be found in ModelDB, Accession numbers 223144, 223269, 223273, and 223274 (senselab.med.yale.edu/modeldb; Migliore et al., 2003; Hines et al., 2004). We checked if we were able to reproduce the original results given in the original publications (see Figure 1 and Table 6). The percentage changes in Table 6 were calculated using:

(20) $$\begin{array}{l} \frac{y-x}{x}\times 100, \end{array}$$

where *x* is the original value and *y* is the reproduced value. We also tested the comparability of the models to each other (see Figures 2–**5**).

**Table 6:** **Model reproducibility**.

**Model**	**Overall reproducibility**	**Variable**	**Original figure**	**Dynamical reproducibility**	**Min %**	**Max %**
De Pittà et al., 2009	++	Ca^2+^	Figure 12a AM	Yes	−4	+5
		IP_3_	Figure 12b AM	Yes	+3	−4
		Ca^2+^	Figure 12a FM	No	−5	−3
		IP_3_	Figure 12b FM	No	−64	−34
Dupont et al., 2011	−/++	Ca^2+^	Figure 2a (blue)	Yes	+21	+34
		DIM	Figure 2a (red)	Yes	−38	+10
		DAG	Figure 2b (blue)	Yes	−30	+17
		PKC	Figure 2b (red)	Yes	−2	+5
		Ca^2+^	Figure 3 (blue)	No	+24	+6
		DIM	Figure 3 (red)	No	+5	+34
Lavrentovich and Hemkin, 2008	+++	Ca^2+^	Figure 3	Yes	0	0
		Ca^2+^	Figure 4 (black)	Yes	+15	+1
		Ca^2+^	Figure 4 (red)	Yes	0	+3
		Ca^2+^	Figure 4 (green)	Yes	0	−6
		Ca^2+^	Figure 4 (blue)	Yes	0	0
		Ca^2+^	Figure 5a	Yes	−1	+1
		Ca^2+^	Figure 5b	Yes	0	+54
		Ca^2+^	Figure 5c	Yes	−3	+76
		Ca^2+^	Figure 7 (black)	Yes	+16	0
		Ca^2+^	Figure 7 (red)	Yes	+13	−12
		Ca^2+^	Figure 9	Yes	+22	0
Riera et al., 2011a,b	−/+/+++	Ca^2+^	Figure 4b (top, black)	Yes/Yes	−36/−23	−41/−3
		*h*	Figure 4b (top, black)	No/Yes	+6/−1	−1/−1
		IP_3_	Figure 4b (top, black)	No/Yes	−99/+1	−10/−4
		Ca^2+^	Figure 4b (top, red)	Yes/Yes	−36/−22	−58/−17
		*h*	Figure 4b (top, red)	No/Yes	+12/+3	−1/−1
		IP_3_	Figure 4b (top, red)	No/Yes	−100/+1	−18/−18

*This table shows how well we were able to reproduce the results of the four selected original publications. The table presents the overall reproducibility of each model, the variables plotted in the original figures, the details of the original figures, dynamical reproducibility (that is, an evaluation of the similarity of the original and reproduced curves), and the change of the original and reproduced curves at minimum and maximum values in percentages. The percentage changes were calculated using Equation (20). For the overall reproducibility, we used our own subjective evaluation (+ means here that about one third was reproduced, ++ means that about two thirds was reproduced, +++ means that all was reproduced, and − means that none of the important features were reproduced). We were able to reproduce about two thirds of the original results by De Pittà et al. (2009). We were not able to reproduce any of the important features of the original results by Dupont et al. (2011) with the original equations, but when we modified one of the equations we were able to reproduce about two thirds of the original results (−/++, see Table 5 for details). We were able to reproduce all of the original results by Lavrentovich and Hemkin (2008) with the help of the corrigendum. We were not able to reproduce any of the important features of the original results by Riera et al. (2011a,b) with the original equations, but when we modified one of the equations we were able to reproduce about one third of the original results when X_IP3_ was 0.43 μM/s between 100 and 900 s and 0 otherwise and all of the original results when X_IP3_ was 0.43 μM/s between 100 and 900 s and 0.2 μM/s otherwise (−/+/+++, see Table 3 for details). For the model by Riera et al. (2011a,b), there are two alternatives for the dynamical reproducibility and percentage changes. The first is when X_IP3_ was 0.43 μM/s between 100 and 900 s and 0 otherwise, and the second is when X_IP3_ was 0.43 μM/s between 100 and 900 s and 0.2 μM/s otherwise*.

## 3. Results

In this study, we chose four single astrocyte models (Lavrentovich and Hemkin, 2008; De Pittà et al., 2009; Dupont et al., 2011; Riera et al., 2011a,b) to test their reproducibility in detail. Additionally, we tested the comparability of pairs of these models in different stimulation conditions or research settings. Table 1 presents a general overview of these studied models and lists our findings on the following six items: Is the model available online, is a graphical illustration of the model given in the original publication, and are all the equations, stimuli, parameter values, and initial conditions given in the original publication. On a closer look, it was also possible to find errors in equations or parameter values. In Tables 2–5, we show the original and modified versions of the equations, initial conditions, and parameter values for the selected four models used in this study. In Table 6, we show how well we were able to reproduce the original results with the information given in the original publication (see also Manninen et al., in press). The table presents the overall reproducibility of each model, the variables plotted in the original figures, the details of the original figures, dynamical reproducibility (that is, an evaluation of the similarity of the original and reproduced curves), and the change of the original and reproduced curves at minimum and maximum values in percentages.

### 3.1. Reproducibility

Lavrentovich and Hemkin (2008) and Riera et al. (2011a,b) studied spontaneous Ca^2+^ oscillations in a single astrocyte model. Lavrentovich and Hemkin (2008) explicitly presented all the equations, parameter values, and initial conditions in their publication and they have additionally provided a corrigendum (see Tables 1, Table 2 for details). They showed five simulation result figures where the variables were plotted against time. We were able to reproduce well all of them (Figures 3–5, 7, 9 of the original publication) with our implementation of the model (see also Manninen et al., in press). The first column from the left of Figure 1 (under “Lavrentovich”) shows the same behavior as Figure 3 of the original publication by Lavrentovich and Hemkin (2008) when using the information in the corrigendum (see Table 6 for more details). It was difficult to extract the exact maximum value from the original figures (Figures 5b,c of the original publication by Lavrentovich and Hemkin, 2008) if the maximum value occurred in an early stage of the simulation. Thus, Table 6 shows large percentage changes when the original and reproduced values are compared.

Riera et al. (2011a,b) presented all model equations and parameter values in their publication (see Tables 1, 3 for details). However, they did not give the initial conditions for the variables, but we were able to obtain them from the results of the original publication (Riera et al., 2011a, see Tables 1, 3 for details). For the Ca^2+^ and IP_3_ concentrations, we set the initial values to 0.09 μM and 0.14 μM, respectively. For the fraction of active IP_3_Rs, we set the initial value to 0.79. The total free Ca^2+^ concentration we set to a constant value of 2 μM (the sum of fluxes over the cell membrane was zero; *j*_in_+*v*_CCE_−*v*_OUT_ = 0) based on Figure 4b of the original publication by Riera et al. (2011a). While trying to reproduce the simulation results as in Figure 4b of the original publication, we realized that there was a typographical error in the original differential equation for the fraction of active IP_3_Rs (see Tables 3, 6 for details). After modifying the equation accordingly, we were able to reproduce, with our implementation of the model, more similar results as in Figure 4b of the original publication. The second column from the left of Figure 1 (under “Riera”) shows that our values for *h* and IP_3_ did not stay high in the beginning of the simulation as the black curves did in Figure 4b of the original publication when *X*_IP3_ was 0.43 μM/s between 100 s and 900 s and 0 otherwise (curves with dotted lines in Figure 1). Especially, the concentration of IP_3_ dropped nearly to zero which can be seen in Table 6 as very high percentage changes in the minimum values. One possible reason for the differing original and reproduced results is that Riera et al. (2011a) must have used a nonzero value for *X*_IP3_ in the beginning of the simulation. Thus, a pulse function of 0.43 μM/s between 100 and 900 s, and otherwise 0.2 μM/s produced about the same curves as the original figure (see curves with solid lines in Figure 1 and Table 6).

De Pittà et al. (2009) and Dupont et al. (2011) modeled neurotransmitter-evoked Ca^2+^ excitability. De Pittà et al. (2009) presented all the equations and parameter values in their publication (see Tables 1, 4 for details). However, they did not give the initial conditions for the variables. For Ca^2+^ concentration, IP_3_ concentration, and the fraction of active IP_3_Rs, we set the initial values to 0.09 μM, 0.22 μM, and 0.78, respectively. De Pittà et al. (2009) showed one simulation result figure (Figure 12 of the original publication with both the amplitude modulation (AM) and frequency modulation (FM)), where the variables were plotted against time. We were able to reproduce well Figure 12 AM of the original publication with our implementation of the model (see the second column from the right of Figure 1 under “De Pittà”). The stimulus used in Figure 1 was a two-pulse wave with alternating Glu concentrations of 2 nM and 5 μM, pulse duration of 62.5 s, and period 125 s. Compared to Figure 12 FM of the original publication, we were not able to reproduce the lower amplitude oscillations toward the end of stimulus and our IP_3_ concentration had smaller values (see Table 6 for details). They have also provided an erratum. However, the erratum did not provide any such information that helped us to reproduce the results.

Dupont et al. (2011) presented a model for mGlu5R-induced Ca^2+^ oscillations. Dupont et al. (2011) presented all the equations and parameter values in the original publication, but did not give the initial conditions for the variables (see Tables 1, 5 for details). For four of the variables, we were able to obtain the initial values from the results of the original publication. The initial values we used were 0.1 μM for the Ca^2+^ concentration, 14 nM for the concentration of DIM, 25 nM for the DAG concentration, and 0.2 for the fraction of active PKC. For the IP_3_ concentration and fraction of Ca^2+^-inhibited IP_3_Rs we decided to use 0.2 μM and 0.9898, respectively (see also Manninen et al., in press). Dupont et al. (2011) presented two figures where the variables were plotted against time. With the original parameter values, we were able to reproduce the oscillating behavior as seen in Figure 2 of the original publication for the concentrations of DAG and DIM, and fraction of active PKC. However, in our implementation, the Ca^2+^ concentration oscillated with very small amplitude (nM). In addition, with the original parameter values we were not able to obtain oscillating Ca^2+^ behavior as in Figure 3 of the original publication. We then checked the references mentioned by Dupont et al. (2011), and decided in this study to change the equation for Ca^2+^ concentration. We modified the equation to be more similar to the one in the publication by Dupont and Croisier (2010) (see Table 5 for details). With this modified model we were able to reproduce the oscillating behavior as in Figure 2 of the original publication by Dupont et al. (2011) (see the first column from the right of Figure 1 under “Dupont” and Table 6 for details). In this case, the stimulus was a constant Glu concentration of 8 μM. When comparing our simulation results to Figure 3 of the original publication, the modified model implemented by us produced more frequent oscillations for Ca^2+^ concentration compared to the original model and the concentration of DIM oscillated once before reaching a steady-state value (see Table 6 for details). We therefore conclude that our modified Ca^2+^ equation was not exactly the same that Dupont et al. (2011) must have used in their original simulations.

### 3.2. Comparability

It was difficult to compare the models by Lavrentovich and Hemkin (2008) and Riera et al. (2011a,b) because these models originally had quite differing dynamical behavior (see Figure 1). However, these models actually have some components that are identical or just have different parameter values (Tables 2, 3); Ca^2+^ efflux from the cytosol to the extracellular space ($v_{out}=k_{out}{\left[Ca^{2+}\right]}_{cyt}$), flow of Ca^2+^ from the extracellular space to the cytosol (parameters *v*_in_ by Lavrentovich and Hemkin, 2008 and *j*_in_ by Riera et al., 2011a,b), and transport of Ca^2+^ from the cytosol to the ER via SERCA pump (*v*_SERCA_). The production and degradation terms of IP_3_ are also almost identical with just different parameter values except that the model by Riera et al. (2011a,b) has two production terms, the parameter *X*_IP3_ in addition to the production term depending on Ca^2+^ concentration. Different equations are used for CICR via IP_3_Rs (named *v*_CICR_ by Lavrentovich and Hemkin, 2008 and *v*_Rel_ by Riera et al., 2011a,b), in which Ca^2+^ is released from the ER to the cytosol. Lavrentovich and Hemkin (2008) and Riera et al. (2011a,b) modeled the leak flux from the ER to the cytosol due to concentration gradient with similar equations but different parameter values. Riera et al. (2011a,b) modeled it as part of the equation for *v*_Rel_. In addition, Riera et al. (2011a,b) modeled the capacitative Ca^2+^ entry (*v*_CCE_) from extracellular space to the cytosol and also had the fraction of active IP_3_Rs as a model variable. Lavrentovich and Hemkin (2008) did not take into account the ratio of effective volumes for cytoplasmic and ER Ca^2+^ in their model.

We tested the model by Lavrentovich and Hemkin (2008) when the sum of ionic fluxes across the cell membrane was zero ($v_{in}-k_{out}\left[Ca_{cyt}^{2+}\right]=0$) and otherwise the same setup as in Figure 1 (see the first column from the left of Figure 2 under “Lavrentovich”). Mimicking this setup in the model by Riera et al. (2011a,b) (compare to the second column from the left of Figure 1 under “Riera”), we changed the parameter producing IP_3_ (*X*_IP3_) to zero in the model by Riera et al. (2011a,b) in addition to having the total free Ca^2+^ concentration as a constant value of 2 μM as in Figure 1 (see the second column from the left of Figure 2 under “Riera”). Comparing these two columns of Figure 2, it is evident that the model by Lavrentovich and Hemkin (2008) has higher Ca^2+^ and IP_3_ concentrations compared to the model by Riera et al. (2011a,b). However, when taking into account the ratio of effective volumes for cytoplasmic and ER Ca^2+^ (β = 35) from the model by Di Garbo et al. (2007) to the model by Lavrentovich and Hemkin (2008), the Ca^2+^ and IP_3_ concentrations became lower than compared to the condition when not taking the ratio into account (not shown). Including this ratio did not work directly with the original setup of the model since model variables ceased to oscillate.

Next, we attempted to maximize the frequency of oscillations in the model by Lavrentovich and Hemkin (2008) to match better the results of the model by Riera et al. (2011a,b). The second column from the right of Figure 2 (under “Lavrentovich”) shows the results when the parameter *V*_M2_ related to the SERCA pump was changed to 5.8 μM/s in the model by Lavrentovich and Hemkin (2008) and a simulation setup otherwise similar as in Figure 1 was used. With this value we were able to obtain more frequent Ca^2+^ oscillations compared to the original attempt presented in Figure 1. The first column from the right of Figure 2 (under “Riera”) shows the results of a setup where the total free Ca^2+^ concentration was a variable and *X*_IP3_ was a constant value of 0.43 μM/s in the model by Riera et al. (2011a,b) and otherwise the simulation setup was similar to Figure 1. It can thus be concluded that these two models have very differing dynamical behavior.

We also tested how the models by Lavrentovich and Hemkin (2008) and Riera et al. (2011a,b) behaved with each others' parameter values when we had net ionic fluxes over the cell membrane (not shown). We studied the equations of both models and decided to change only those parameter values that were in equations of exactly identical form in both models (parameters *v*_in_ vs. *j*_in_, *v*_M2_ vs. *V*_SERCA_, *k*_f_ vs. *v*_2_, *v*_p_ vs. *v*_δ_, *k*_p_ vs. *K*_δCa_, and *k*_deg_ vs. *K*_IP3_ by Lavrentovich and Hemkin (2008) and Riera et al. (2011a,b), respectively). We tested both modifying all values simultaneously, and modifying them one by one. We discovered that the model by Lavrentovich and Hemkin (2008) was not able to oscillate at all or only once in 600 s with any of the values by Riera et al. (2011a,b), neither when parameters were tested one by one nor when they were tested simultaneously. When testing the model by Riera et al. (2011a,b) with the parameter values of the model by Lavrentovich and Hemkin (2008), we found out that if the two values for the same parameter were almost similar, the model by Riera et al. (2011a,b) still oscillated with the parameter value from the model by Lavrentovich and Hemkin (2008). *X*_IP3_ would appear to be the most important parameter causing the model by Riera et al. (2011a,b) to oscillate. If *X*_IP3_ was zero, the model did not oscillate with the original parameter value or with any parameter value from the model by Lavrentovich and Hemkin (2008).

We compared the model by De Pittà et al. (2009) and our modified version of the model by Dupont et al. (2011) using four different stimuli. Figure 3 shows the model behaviors when the stimuli were two different constant Glu concentrations. The two columns from the left of Figure 3 show how the models behaved when the stimulus was a constant Glu concentration of 0.1 μM. We chose this stimulus because both models oscillated with a value this small. The two columns from the right of Figure 3 show how the models behaved when the stimulus was a constant Glu concentration of 2.5 μM. This stimulus was chosen because it clearly brought out the difference between these two models. The simulation results of the model by De Pittà et al. (2009) with a constant Glu stimulus of 2.5 μM showed how all the model variables, Ca^2+^, IP_3_, and fraction of active IP_3_Rs, oscillated, whereas the simulation results of our modified version of the model by Dupont et al. (2011) showed oscillations with only two model variables, Ca^2+^ concentration and the fraction of Ca^2+^-inhibited IP_3_Rs. In addition, it should be noted that the models had opposite behaviors with these two stimulus values; the higher stimulus value produced higher Ca^2+^ concentrations with the model by De Pittà et al. (2009), but it produced lower Ca^2+^ concentrations with our modified version of the model by Dupont et al. (2011). Based on experimental data (Honsek et al., 2012; Haustein et al., 2014), the Ca^2+^ concentration is higher when the Glu concentration is higher, and the model by De Pittà et al. (2009) seems to behave more realistically than our modified version of the model by Dupont et al. (2011) in this sense (see Figure 3).

Figure 4 shows model dynamics when the Glu stimuli were two different seven-pulse waves. The two columns from the left of Figure 4 show how the models behaved when the Glu stimulus was a seven-pulse wave with alternating concentrations of 2 nM and 5 μM, pulse duration of 5 s, and period 15 s. The two columns from the right of Figure 4 show how the models behaved when the Glu stimulus was a seven-pulse wave with alternating concentrations of 2 nM and 5 μM, pulse duration of 1 s, and period 6 s. In our modified version of the model by Dupont et al. (2011), the Ca^2+^ concentration oscillated even with the Glu concentration of 2 nM, which was not the case with the model by De Pittà et al. (2009) (see Figure 4). The model by Dupont et al. (2011) was developed and tested for a constant stimulus, whereas the model by De Pittà et al. (2009) was developed for a varying stimulus (see Figures 3–5).

Since the model by De Pittà et al. (2009) and our modified version of the model by Dupont et al. (2011) produced opposite results, we decided to investigate their dynamical behavior in more detail. Our modified version of the model by Dupont et al. (2011) did not oscillate with all the model variables when the stimulus was a constant Glu concentration between 1.8 μM and 3.4 μM or zero. We also discovered that when the stimulus was a constant Glu concentration higher than 3.8 μM, the model by De Pittà et al. (2009) ceased to oscillate during the simulation, and it reached a steady-state. The higher the constant stimulus concentration, the faster the model by De Pittà et al. (2009) ceased to oscillate. At a constant Glu concentration of 3.8 μM, the model ceased to oscillate around 500 s. At a constant Glu concentration of 4 μM, the model ceased to oscillate around 300 s. At a constant Glu concentration of 8 μM (the original stimulus of the model by Dupont et al., 2011), the model ceased to oscillate around 100 s (see Figure 5). Such a long-lasting constant stimulus may be considered to mimic cell culture conditions where a neurotransmitter is applied with a pipette and not immediately rinsed. We also tested our modified version of the model by Dupont et al. (2011) with the original stimulus of the model by De Pittà et al. (2009) (compare Figures 1 and 5).

## 4. Discussion

In this study, we evaluated four relatively simple computational models of astrocytes (Lavrentovich and Hemkin, 2008; De Pittà et al., 2009; Dupont et al., 2011; Riera et al., 2011a,b) by implementing the equations based on what was presented in the original publications. Our aim was to reproduce the simulation results of the original publications and compare them to see if the models can substitute one another. Unexpectedly, we found out that three of the model publications did not give all the necessary information needed to implement these models (see also Manninen et al., in press). Moreover, we were able to reproduce the original results of only one of the four models completely based on the information in the original publications and errata (Lavrentovich and Hemkin, 2008). We actually found obvious errors in two of the model publications (Dupont et al., 2011; Riera et al., 2011a,b). When we modified the equations, the reimplemented models produced the original results more accurately.

In addition to reproducibility, we also addressed the comparability of the models. Even though these models are assumed to describe relatively similar biological processes, their behaviors are quite different, making it difficult to compare them. The model by Riera et al. (2011a,b) oscillated more frequently than the model by Lavrentovich and Hemkin (2008). We found out that the models by Lavrentovich and Hemkin (2008) and Riera et al. (2011a,b) were sensitive to parameter values, especially the model by Lavrentovich and Hemkin (2008) changed its behavior completely when using the parameter values from the model by Riera et al. (2011a,b). Overall, the simulation results of the model by De Pittà et al. (2009) and our modified version of the model by Dupont et al. (2011) showed similar kind of behavior when a constant stimulus was used. However, a higher stimulus value produced higher Ca^2+^ concentrations with the model by De Pittà et al. (2009), whereas it produced lower Ca^2+^ concentrations with our modified version of the model by Dupont et al. (2011). Furthermore, the higher the constant stimulus concentration, the quicker the model by De Pittà et al. (2009) ceased to oscillate. The two models produced differing results when using the same pulse wave stimuli. The Ca^2+^ concentration oscillated even with a low stimulus concentration in our modified version of the model by Dupont et al. (2011), which was not the case with the model by De Pittà et al. (2009).

We conclude that the four studied models consider only a subset of mechanisms responsible for astrocyte Ca^2+^ excitability and leave out several essential mechanisms, such as the cell membrane ionic currents and various intracellular signaling cascades. Based on these results we are unable to conclude if any of these models is a suitable generic model for astrocyte excitability. However, we conclude that since the dynamical behavior of the models is quite different with the same parameter values or stimulus, they cannot be considered to represent exactly the same astrocyte subtype or phenomena. Future work should include sophisticated validation of computational models with *in vitro* and *in vivo* experimental data.

In neuroscience, reproducibility and comparability of research results have gained a lot of interest over the past years (Teeters et al., 2008; Mochizuki et al., 2016; Zehl et al., 2016). Simultaneously, computational models of brain function are being introduced in a rapidly increasing quantity. Modeling in neuroscience offers a useful tool for integrating current knowledge and producing intelligent hypotheses about mechanisms of brain function on all levels of organization. However, it is a frequent problem that publications lack crucial details in how the models are presented, making it hard to reproduce the original simulation results (see, e.g., Manninen et al., 2010, in press). We have discovered that too often graphical illustrations of the models are misleading or completely missing, and sometimes all equations are not explicitly given in the publications, but are just referred to with a citation to a previous model publication (see, e.g., Manninen et al., 2010, in press). Thus, it is often difficult to know exactly what the actual model components are. The field of computational neuroscience benefits from published, well-documented, and well-validated models with detailed information about the exact biological subsystem the model is developed for. Careful consideration of all the aforementioned points enhances model re-usability in future research and should accelerate the development of more accurate and comprehensive models to decipher various aspects of the functioning of the brain. Due to problems similar to those described in this publication, reproducibility and comparability of research results have recently gained much interest in computational neuroscience, as well as in neuroscience in general.

To promote re-usability of models, several model databases are available to store models and metadata for future use, such as ModelDB (Hines et al., 2004), BioModels database (Le Novère et al., 2006), and the CellML Model Repository (Lloyd et al., 2008). Database systems for both published data and models are being developed by international large-scale projects such as Allen Institute for Brain Science (www.alleninstitute.org) and Human Brain Project (www.humanbrainproject.eu). The Open Source Brain initiative (www.opensourcebrain.org) is an online platform which aims to facilitate sharing and collaborative development of neuronal models. Very few systems, however, address in full detail the reproducibility of the stored models. Part of the challenge is evidently related to funding and resources of reproduction of models. Efficient testing of reproducibility in the publication process requires personnel capable of testing the models, and informatics systems supporting easy, user-friendly testing. As indicated by our study with computational astrocyte models, there is a clear need for publishing platforms that stress reproducibility.

Since the scientific community across all disciplines in bioscience faces the same challenge of ensuring accessibility, reproducibility, and efficient comparability of scientific results, a set of guidelines and good practices should be employed. To promote reproducible science, good model description practices for realistic neuronal network models (Nordlie et al., 2009) have been suggested in addition to minimum information requirements for reproduction (Le Novère et al., 2005; Waltemath et al., 2011a). In addition, many Extensible Markup Language (XML)-based model and simulation representation formats have been developed, such as SBML (Hucka et al., 2003), CellML (Lloyd et al., 2004), NeuroML (Gleeson et al., 2010), SED-ML (Waltemath et al., 2011b), and LEMS (Cannon et al., 2014). Jupyter Notebook (earlier known as IPython Notebook) is a potential technology to enhance reproducibility and accessibility. However, many authors still do not make their models publicly available or they publish their models in a format that is not easily exchangeable between different simulation platforms. These issues should be reflected in the training of young scientists in neuroscience, including computational neuroscientists (see also Akil et al., 2016).

Good practices could be developed and enforced by international neuroscience organizations and publishers to steer the development of the field and to improve the quality of published work as follows. First, more emphasis should be put on presenting a set of figures describing the function of all model variables. The actual model code files and information needed for interpreting them should be made available when publishing a model. In addition, information necessary to reimplement the model and reproduce the original simulation results should be presented. These include, for example, all numerical values of parameters, initial conditions, and stimuli used in each simulation. This will further facilitate model development and reuse, as well as the use of models as educational tools for younger scientists. Finally, reviewers should have the responsibility to request all the above-mentioned information in the publications to ensure the reproducibility of published models.

In summary, we have pointed out several challenges in the field of computational neuroscience, specifically in relation to reproducibility and comparability of computational models, using models of astrocyte Ca^2+^ excitability as examples. The key findings of the present work can be summarized as follows. First, our results stress the importance of proper comparison of models developed for similar phenomena and validation of models against experimental data. Second, our results emphasize a careful, critical review process of the developed models. Third, our work points out that a variety of aspects of model development and presentation could be improved. The style and comprehensiveness of how to present the model details are examples of such crucial aspects. Specifically, all necessary mathematical equations, as well as the parameter values of equations, the initial values of variables, and the stimuli used, should be given precisely. Fourth, model codes should be made publicly available. We expect that ultimately the large-scale, global neuroscience and neuroinformatics projects and initiatives (see, e.g., Markram et al., 2015; Bouchard et al., 2016; Grillner et al., 2016) will help in solving the current challenges in model validation, reproducibility, and comparability.

## Author contributions

Conceived and designed the experiments: TM, RH, and ML. Implemented the models and performed the simulations: TM. Analyzed, evaluated, and compared the results: TM, RH, and ML. Wrote the article: TM, RH, and ML.

## Funding

This project received funding from the European Union Seventh Framework Programme (FP7) under grant agreement No. 604102 (HBP), European Unions Horizon 2020 research and innovation programme under grant agreement No. 720270, and Academy of Finland (decision No. 297893).

### Conflict of interest statement

The authors declare that the research was conducted in the absence of any commercial or financial relationships that could be construed as a potential conflict of interest.
